# Author Correction: A c-di-GMP signaling module controls responses to iron in *Pseudomonas aeruginosa*

**DOI:** 10.1038/s41467-024-52012-2

**Published:** 2024-10-08

**Authors:** Xueliang Zhan, Kuo Zhang, Chenchen Wang, Qiao Fan, Xiujia Tang, Xi Zhang, Ke Wang, Yang Fu, Haihua Liang

**Affiliations:** 1 College of Life Sciences, Northwest University, Xi’an, ShaanXi China; 2 College of Medicine, Southern University of Science and Technology, Shenzhen, China; 3 Department of Pulmonary and Critical Care Medicine, The First Affiliated Hospital of Guangxi Medical University, Nanning, China; 4 University Laboratory of Metabolism and Health of Guangdong, Southern University of Science and Technology, Shenzhen, China

**Keywords:** Bacterial structural biology, Biofilms

Correction to: *Nature Communications* 10.1038/s41467-024-46149-3, published online 29 February 2024

The original version of this Article contained two errors in Fig. 2d, in which the CLSM image of PAO1 in the BIP row was inadvertently duplicated from the LB row of PAO1, and the CLSM image of Δ*ismP* in the Fe^3+^ row was inadvertently duplicated from the BIP row of Δ*ismP*/p-*ismP*. This has been corrected in both the PDF and HTML versions of the Article.

