# Supplementary material for: A c-di-GMP signaling module controls responses to iron in Pseudomonas aeruginosa
Source: Nat Commun. 2024 Feb 29;15:1860. doi: 10.1038/s41467-024-46149-3 (PMC10904736; doi:10.1038/s41467-024-46149-3)
Supplement: Supplementary file 1 — Supplementary information [file 41467_2024_46149_MOESM1_ESM.pdf]

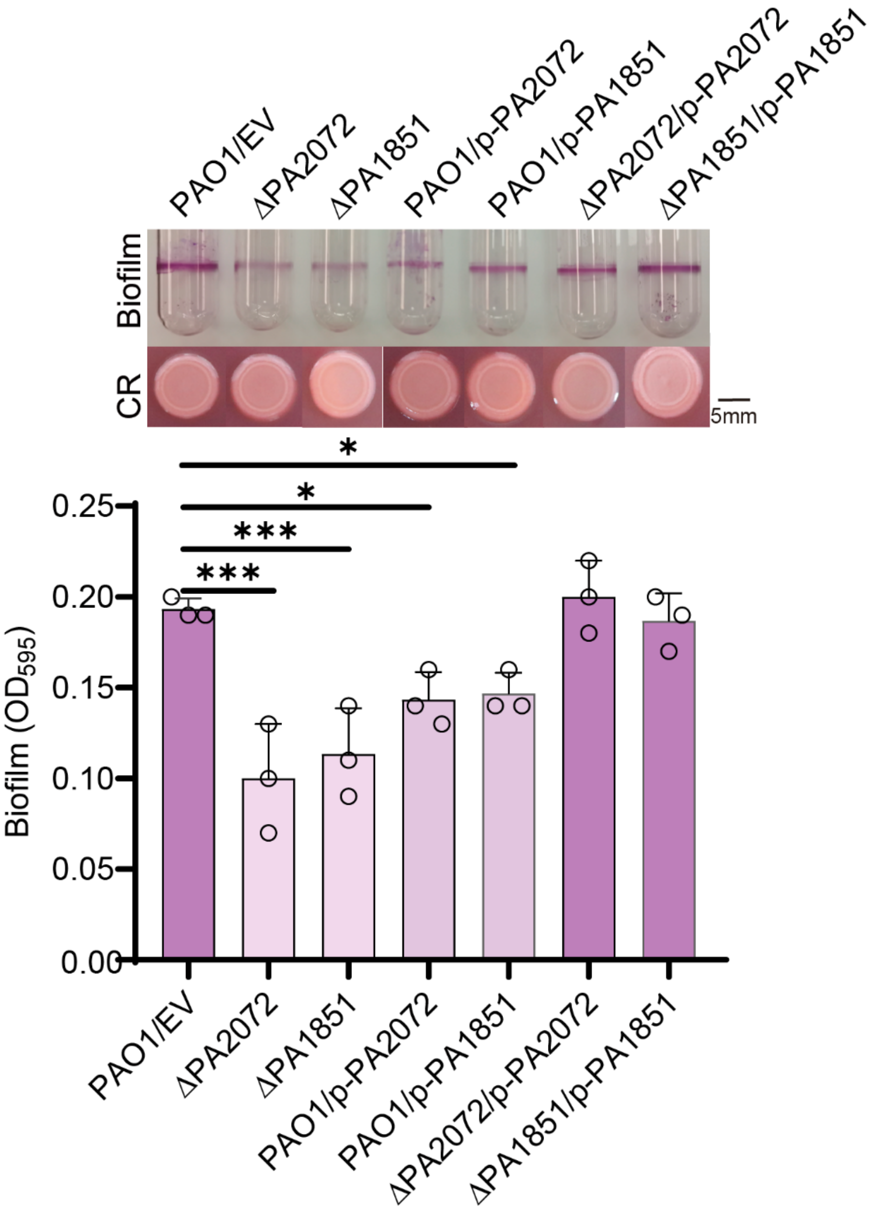

Supplementary Fig. 1 | Impact of PA1851 and PA2072 gene manipulation on biofilm formation. The biofilm of strains cultured for 14 h was stained with crystal violet (**top**) and quantified by measurement of OD_{595} (**bottom**). Colonies morphology of the indicated strains after 2 days of incubation and growth on Congo red plates (mid). Error bars indicate the means ± s.d. of three independent replicates. * $P < 0.05$, *** $P < 0.001$ based on one-way ANOVA test.

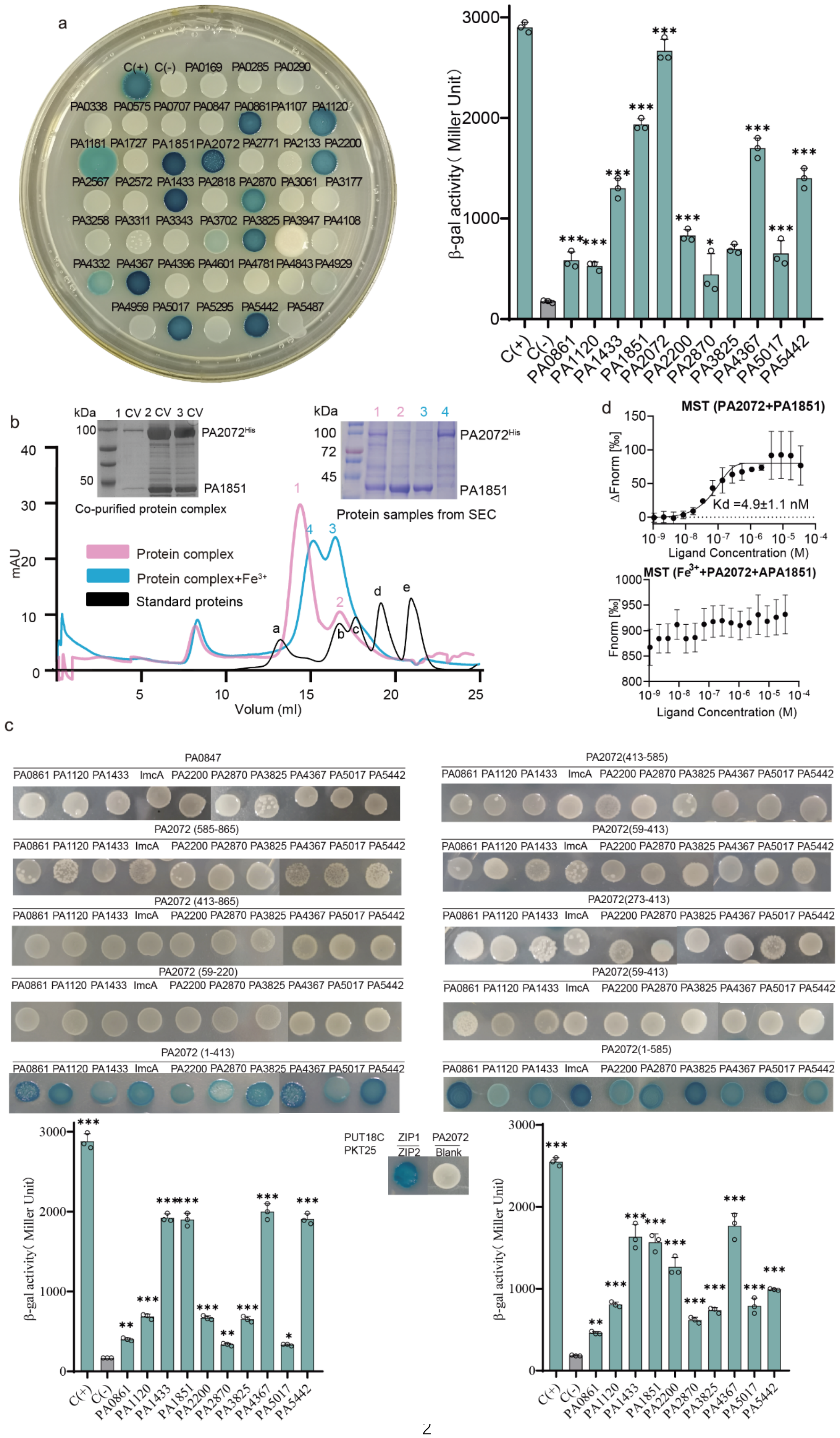
a
C(+) C(-) PA0169 PA0285 PA0290
PA0338 PA0575 PA0707 PA0847 PA0861 PA1107 PA1120
PA1181 PA1727 PA1851 PA2072 PA2771 PA2133 PA2200
PA2567 PA2572 PA1433 PA2818 PA2870 PA3061 PA3177
PA3258 PA3311 PA3343 PA3702 PA3825 PA3947 PA4108
PA4332 PA4367 PA4396 PA4601 PA4781 PA4843 PA4929
PA4959 PA5017 PA5295 PA5442 PA5487
β-gal activity (Miller Unit)
C(+) C(-) PA0861 PA1120 PA1433 PA1851 PA2072 PA2200 PA2870 PA3825 PA4367 PA5017 PA5442
b
kDa 1 CV 2 CV 3 CV
PA2072His
PA1851
Co-purified protein complex
Protein samples from SEC
Protein complex
Protein complex+Fe3+
Standard proteins
mAU
Volum (ml)
d
MST (PA2072+PA1851)
ΔFnorm [‰]
Kd =4.9±1.1 nM
Ligand Concentration (M)
MST (Fe3++PA2072+APA1851)
Fnorm [‰]
Ligand Concentration (M)
c
PA0847
PA2072(413-585)
PA2072 (585-865)
PA2072(59-413)
PA2072 (413-865)
PA2072(273-413)
PA2072 (59-220)
PA2072(59-413)
PA2072 (1-413)
PA2072(1-585)
PA0861 PA1120 PA1433 ImcA PA2200 PA2870 PA3825 PA4367 PA5017 PA5442
PUT18C PKT25
ZIP1 ZIP2
PA2072 Blank
β-gal activity(Miller Unit)

Supplementary Fig. 2 | Specificity of interaction between PA2072 and PA1851. a, BTH assay revealed the interaction between IsmP and 10 proteins involved in c-di-GMP metabolism. **b**, The three lanes in the top-left SDS-PAGE image represent samples from the continuous elution (triple column volumes) of the co-expressed protein complex in M9 medium using buffer containing 300 mM imidazole. And samples from indicated peak of the SEC elution curve (**bottom**) of the PA2072^{his}-PA1851 complex in the absence or presence of Fe^{3+} were separated using SDS-PAGE and shown on the top-right inset. This experiment was independently repeated three times with similar results. The black curve represents the calibration data of the chromatography column (Superdex 6 Increase), plotting relative molecular weight (Mr) against elution volume. Five standard proteins were used: a lyophilized mix of thyroglobulin (670 kDa), bovine γ-globulin (158 kDa), chicken ovalbumin (44 kDa), equine myoglobin (17 kDa), and vitamin B12 (1.35 kDa). By fitting the elution volume against the logarithm of the molecular weight (lgMr), the following regression line equation was obtained: $y=-0.3408x+7.6571$ ($R^2=0.9295$), where y and x represent the lgMr and the elution volume, respectively. The slope of -0.3408 and the intercept of 7.6571 were obtained through data fitting. The relative molecular weights of one DDM micelle molecule, PA1851, and PA2072^{His} are approximately 70 kDa[1], 44.8 kDa, and 98.5 kDa, respectively. Based on the above calculation, the relative molecular weights of the proteins from corresponding SEC elution peak are as follows: peak 1 (~483.9 kDa), peak 2 (~129.0 kDa), peak 3 (~150.3 kDa), and peak 4 (~266.6 kDa) **c**, BTH assay verified the interaction between different domains of PA2072 and 10 other c-di-GMP metabolic enzymes. The quantitative analyses were measured by β-galactosidase activity level (in Miller units) in *E. coli* BTH101 cells (bottom). PA0847 did not interact with the 10 candidate proteins. Error bars indicated the means ± s.d. of three biological replicates. * $P < 0.05$, ** $P < 0.01$, ***$P < 0.001$ based on one-way ANOVA test. **d**, MST analysis showed PA2072^{His} bound to PA1851(**upper**) but PA2072^{His} mixed with 100 μM Fe^{3+} did not bind to PA1851 (**bottom**). Dissociation constants (K_D), expressed in nM, are presented. Error bars indicate the means ± s.d. of three independent replicates.

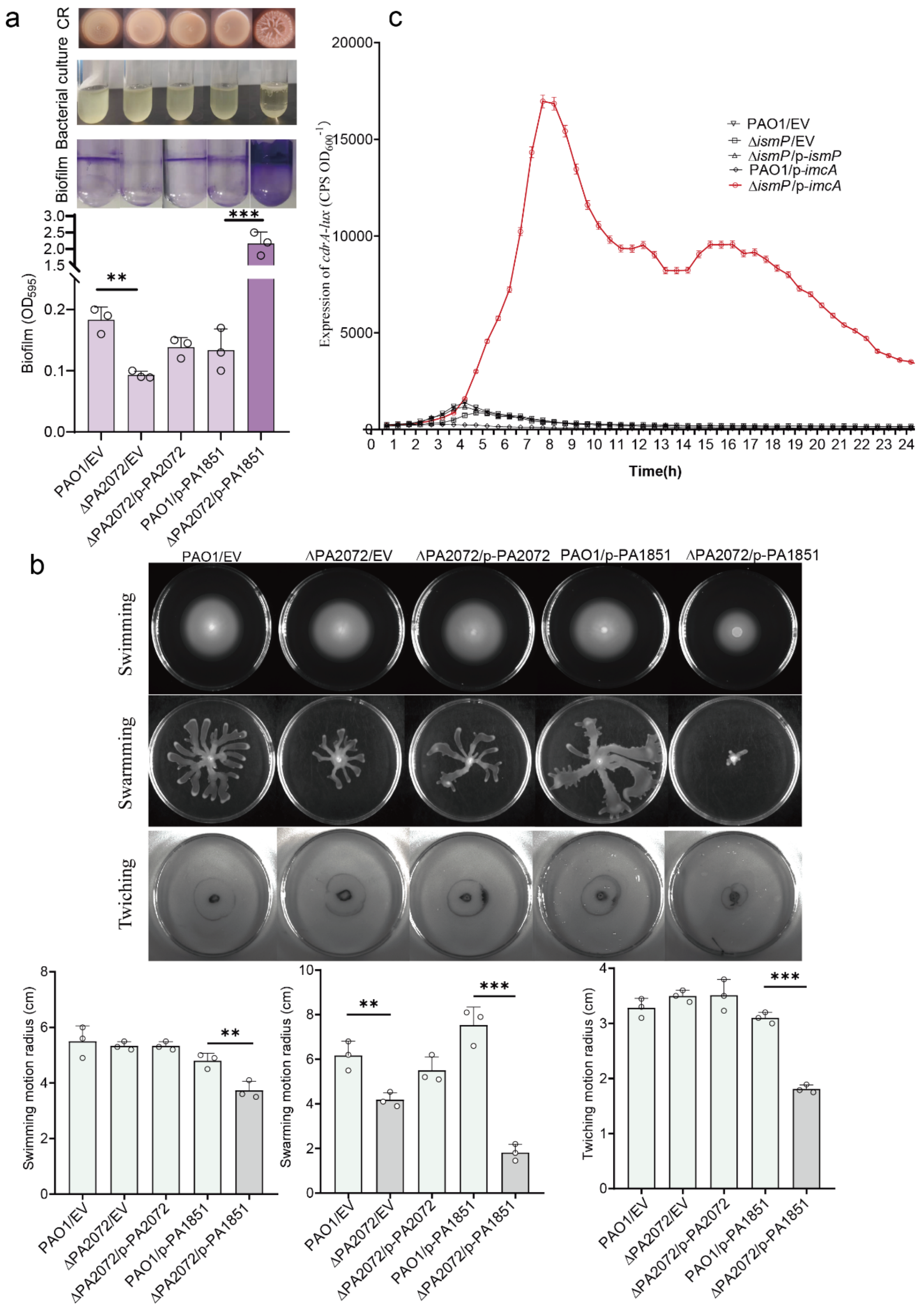

Supplementary Fig. 3 | Biofilm formation and bacterial motility of the indicated strains. a, Cellular aggregation (**up**), and biofilm formation of the indicated strains, grown in LB for 20 h, was displayed with crystal violet staining (mid) and quantified with optical density measurement (**down**). **b**, Expression of *imcA* in Δ*ismP* mutant inhibited bacterial motility. **c**, The expression of *cdrA-lux*

was measured in the indicated strains. Error bars indicated the means ± s.d. of three independent replicates. $**P < 0.01$, $***P < 0.001$ based on one-way ANOVA test.

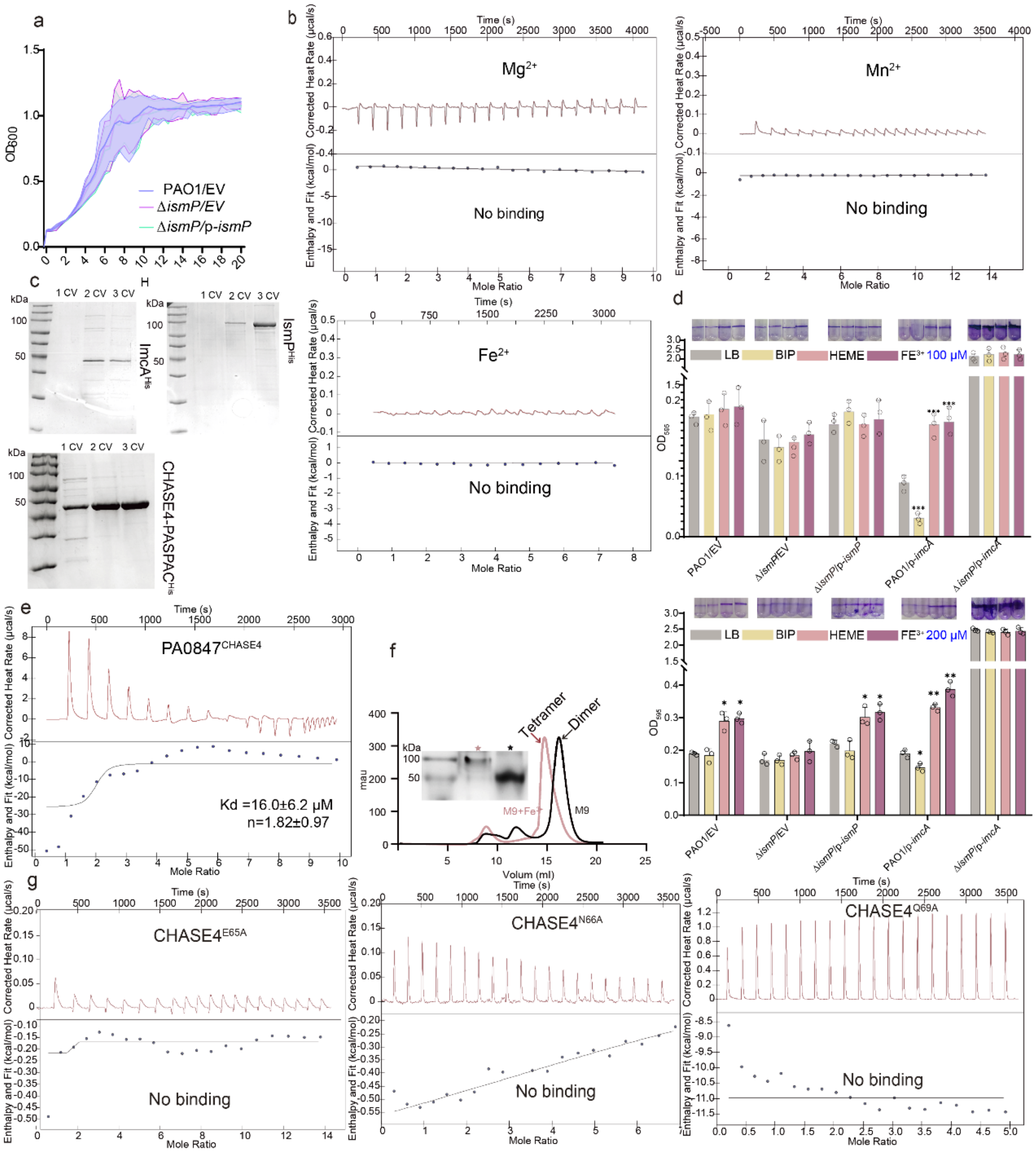

Supplementary Fig. 4 | CHASE4 binding with different metal ions and its role in biofilm formation. **a**, The growth curves of PAO1, Δ*ismP* and Δ*ismP*/p-*ismP* in LB medium. **b,** ITC showing the binding of the CHASE4 domain with Mn^{2+}, Mg^{2+}, and Fe^{2+}. Upper panel shown the exothermic peaks that correspond to 20 injections (4 μL per injection) of 500 μM Mn^{2+}, Mg^{2+}, and Fe^{2+} solution into 55 μM protein performed at 175 second intervals. To derive the dissociation constant, the binding isotherm (lower panel) of the normalized heats as a function of the molar ratio was fit to a single-binding-site model using the Origin software package. **c**, SDS-PAGE results showcased the individual proteins that had been purified. This experiment was independently repeated three times with similar results. Three lanes represent the continuous elution of triple column volumes of samples using elution buffer containing 300 mM imidazole. **d**, After adding compounds to the culture medium and statically incubating the bacterial culture, a stain with crystal violet was applied. The OD_{595} was measured to quantify the biofilm. **e**, ITC assessed the binding of the CHASE4 domain to iron in PA0847. One-way Analysis of Variance (ANOVA) using a Tukey's multiple comparisons test was used for significance testing. Values are means ± s.d. (n=3); $*P < 0.05$, $**P < 0.01$. **f**, SEC showed that CHASE4 changed from a dimer to tetramer under iron-rich conditions. In the SEC analysis, the elution volumes for the tetrameric and dimeric forms of the protein were observed at 14.5 mL and 16.3 mL, respectively. The relative molecular weight of samples from corresponding SEC elution peak were identified using native-PAGE and shown in the inset. **g**, ITC revealed that no binding was observed between iron and these CHASE4 mutants. These experiments were repeated at least three times, and similar results were obtained.

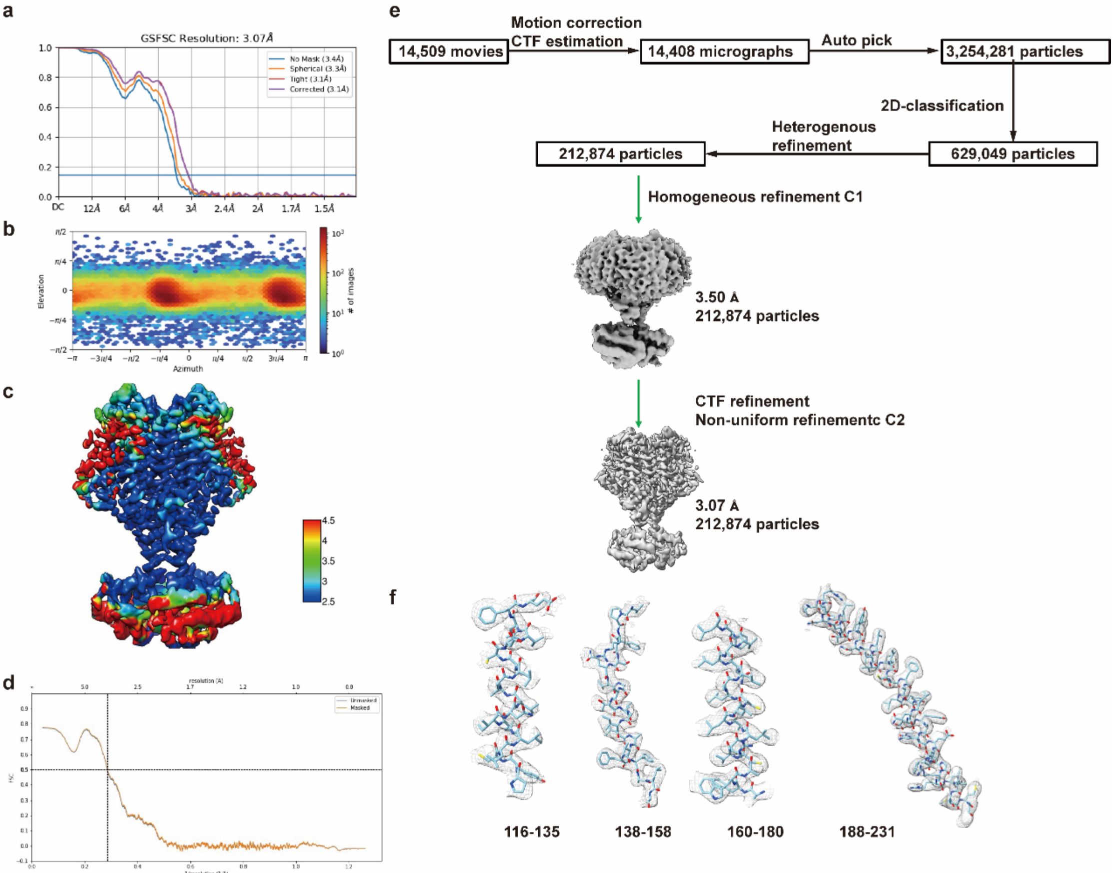

Supplementary Fig. 5 | Cryo-EM analysis of ImcA in omplex with substrate analog GMPCPP. **a-d,** are FSC curves, the viewing direction distribution plot, local resolution map and model-to-map

FSC of GMPCPP-bound ImcA homodimer, respectively. **e,** Flowchart for cryo-EM data processing. **f,** Representative cryo-EM density maps (2Fo-Fc, σ=12).–

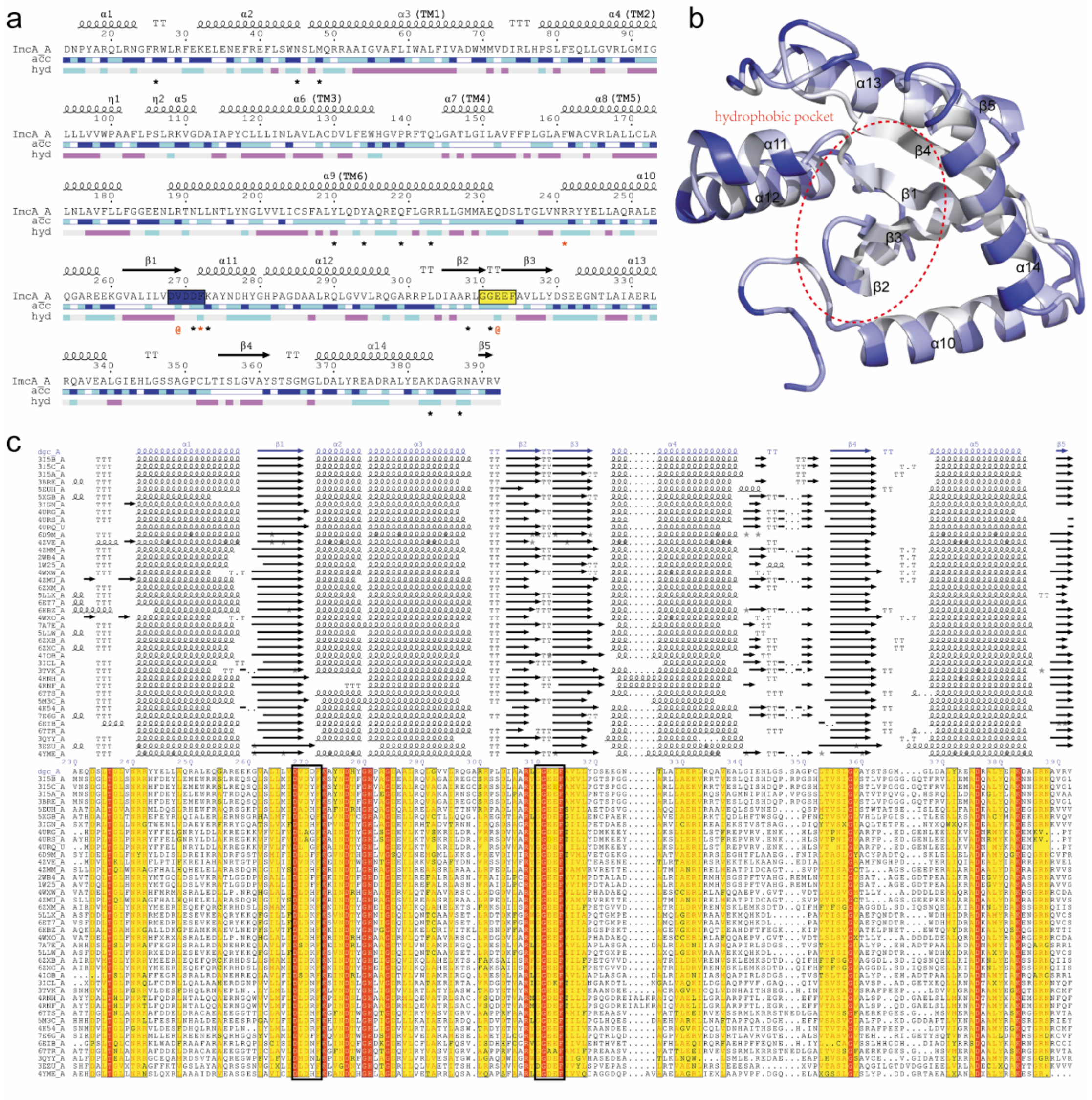

Supplementary Fig. 6 | ImcA DGC domain have two relatively conserved motifs, D-riched ^{68}'DVDDF^{72}' motif and ^{301}'GGEEF^{315}' motif and a hydrophobic ligand binding pocket. a, The sequence analysis of DGC protein ImcA with corresponding secondary structural units defined by DSSP algorithm indicated above. D-riched ^{68}'DVDDF^{72}' motif and ^{301}' GGEEF^{315}' motif are colored in blue and yellow with black outline, respectively. **b**, The cartoon diagram of the GGDEF domain located in the DGC protein ImcA. The hydrophobic ligand pocket, which primarily consists of spatially consecutive β-sheets together with α10 and α14 helixes, is encircled by a red ellipse dotted line. **c**, The multiple sequence alignment of ImcA with its homologs deposited in PDB with corresponding secondary structural units that defined by DSSP algorithm displayed successively above. The conserved core motifs are surrounded by rectangular black solid lines.

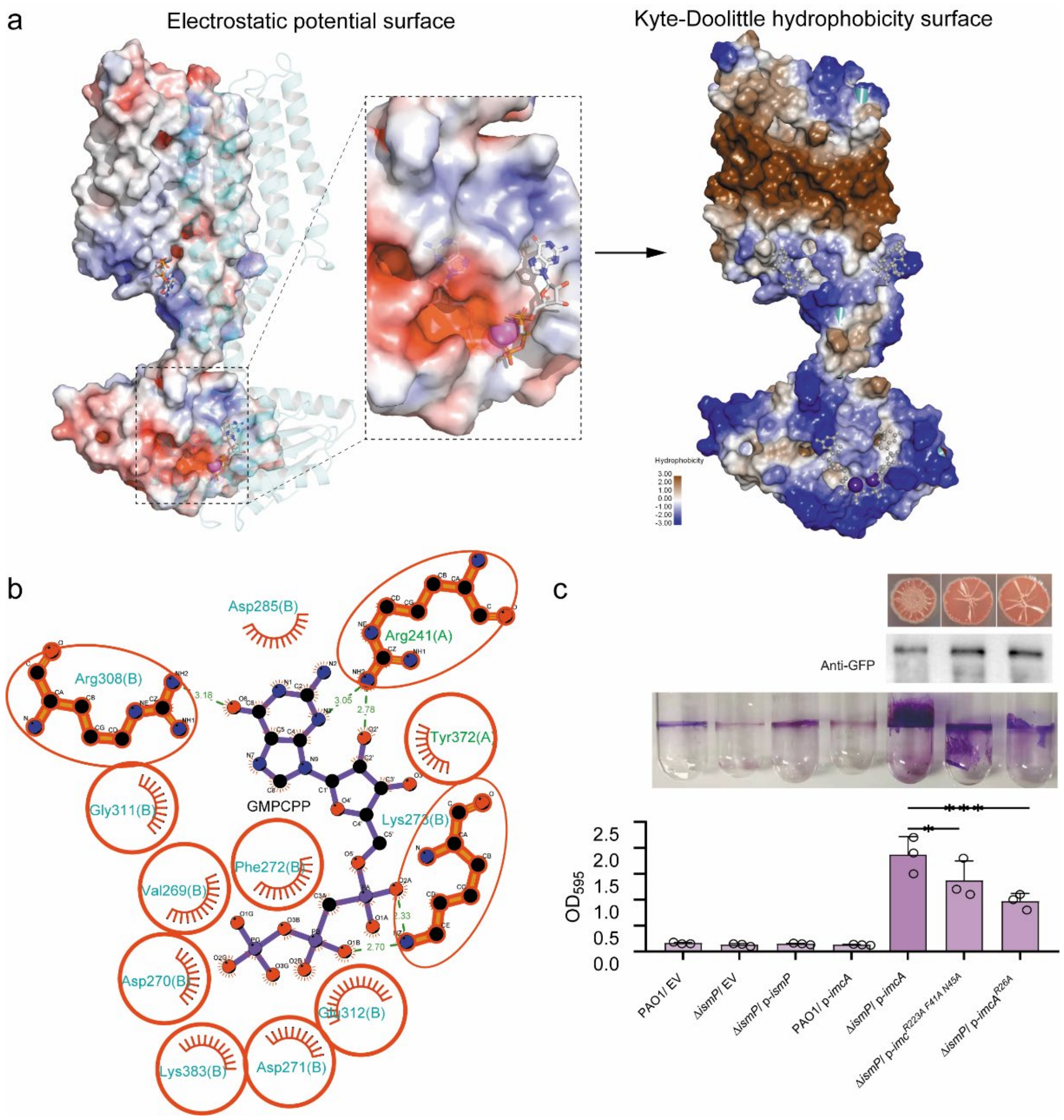

Supplementary Fig. 7 | ImcA have the ability to enrich substrate molecules to the inner membrane-proximal vicinity. a, The electrostatic potential surface, which is generated by the APBS electrostatics plugin in PyMOL, of ImcA and the enlarged details of its substrate binding pocket located in the DGC domain. **b**, The diagram of interaction details observed in the membrane-proximal GMPCPP binding pockets, and residues referring to two pockets are encircled by a red ellipse (drawn by LigPlot$^+$ v.2.2). The residue labels are composed successively of the three-letter identifier of certain amino acid, corresponding residue number and chain name in parentheses. **c**, Rresidues located in the vicinity of membrane-proximal GMPCPP binding pockets were critical for biofilm formation and colonies morphology. Western blot showed that these two mutants were expressed as wt CHASE4. Error bars indicate the means ± s.d. (n=3) of three biological replicates. *** $P < 0.001$ based on one-way ANOVA test.

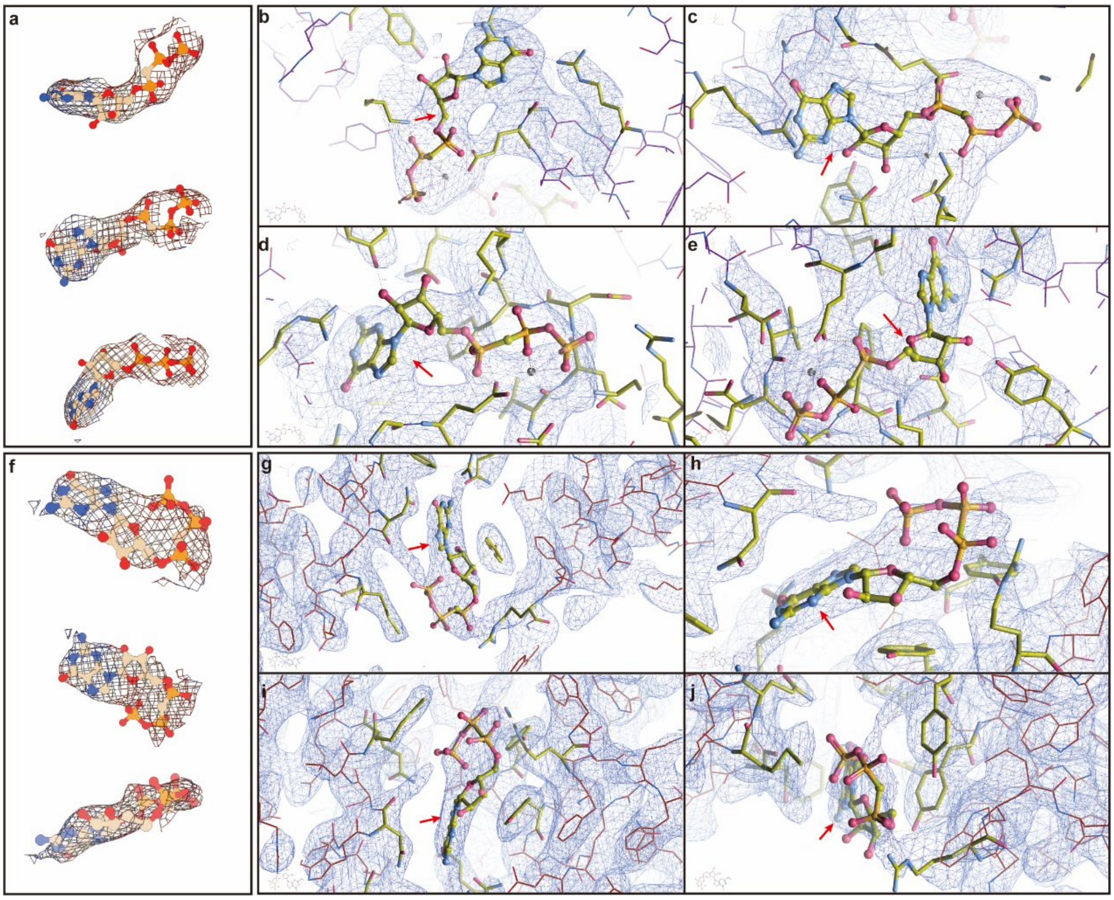

Supplementary Fig. 8 | The CryoEM electron density of GMPCPP ligands in ImcA dimer.
Several views of GMPCPP ligands located in the catalytic pocket (a-e) and membrane-proximal cytoplasmic side, respectively (f-j), of ImcA with corresponding CyroEM electron density maps shown in mesh style (σ=6.5). Given the symmetry, only one of the symmetrically equivalent mates will thereby be displayed here. Figures were drawn by ChimeraX or Coot.

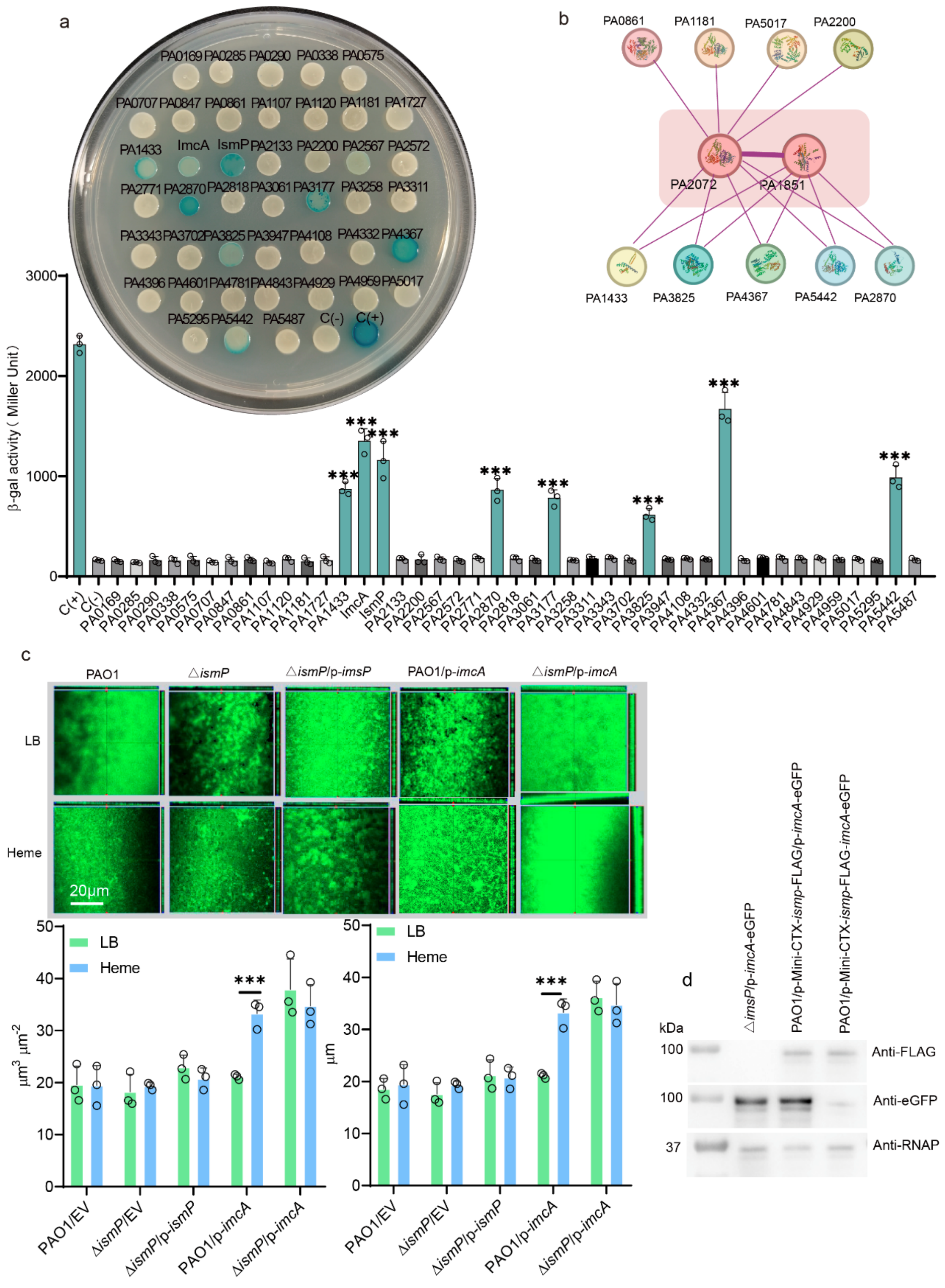

Supplementary Fig. 9 | Interactions of ImcA with c-di-GMP metabolic enzymes (a, b), quantification of biofilm formation (c), and Western Blot analysis of *imcA*-eGFP and *ismP*-FLAG (d). a, the interaction between the ImcA and 43 proteins related to c-di-GMP metabolism was evaluated by BTH assay. **b**, The interacting network of both ImcA and Ismp with other c-di-GMP enzymes based on our BTH results. **c**, Confocal Laser Scanning Microscopy (CLSM) measured biofilms grown in various medium conditions. In biofilm images, the flow was oriented from left to right, with scale bars of 20 μm. Biomass and thickness were measured in $\mu m^3\ \mu m^{-2}$ and

µm. One-way Analysis of Variance (ANOVA) using a Tukey's multiple comparisons test was used for significance testing. Error bars indicate means ± s.d. (n=3); *$P < 0.05$, **$P < 0.01$, *** $P < 0.001$. **d**, Western blot analysis of ImcA-eGFP and IsmP-Flag in wild-type PAO1, PAO1/p-*imcA*-eGFP, and Δ*ismp*/p-*imcA*-eGFP strains after 20 h of cultivation. The Mini-*imcA*-eGFP and/or Mini-*ismP*-FLAG were integrated into the chromosome of the indicated strains. This experiment was independently repeated three times with similar results. Samples of bacterial pellets from the indicated strains were blotted with anti-eGFP, anti-FLAG, and anti-RNAP antibodies. RNAP was used as a loading control.

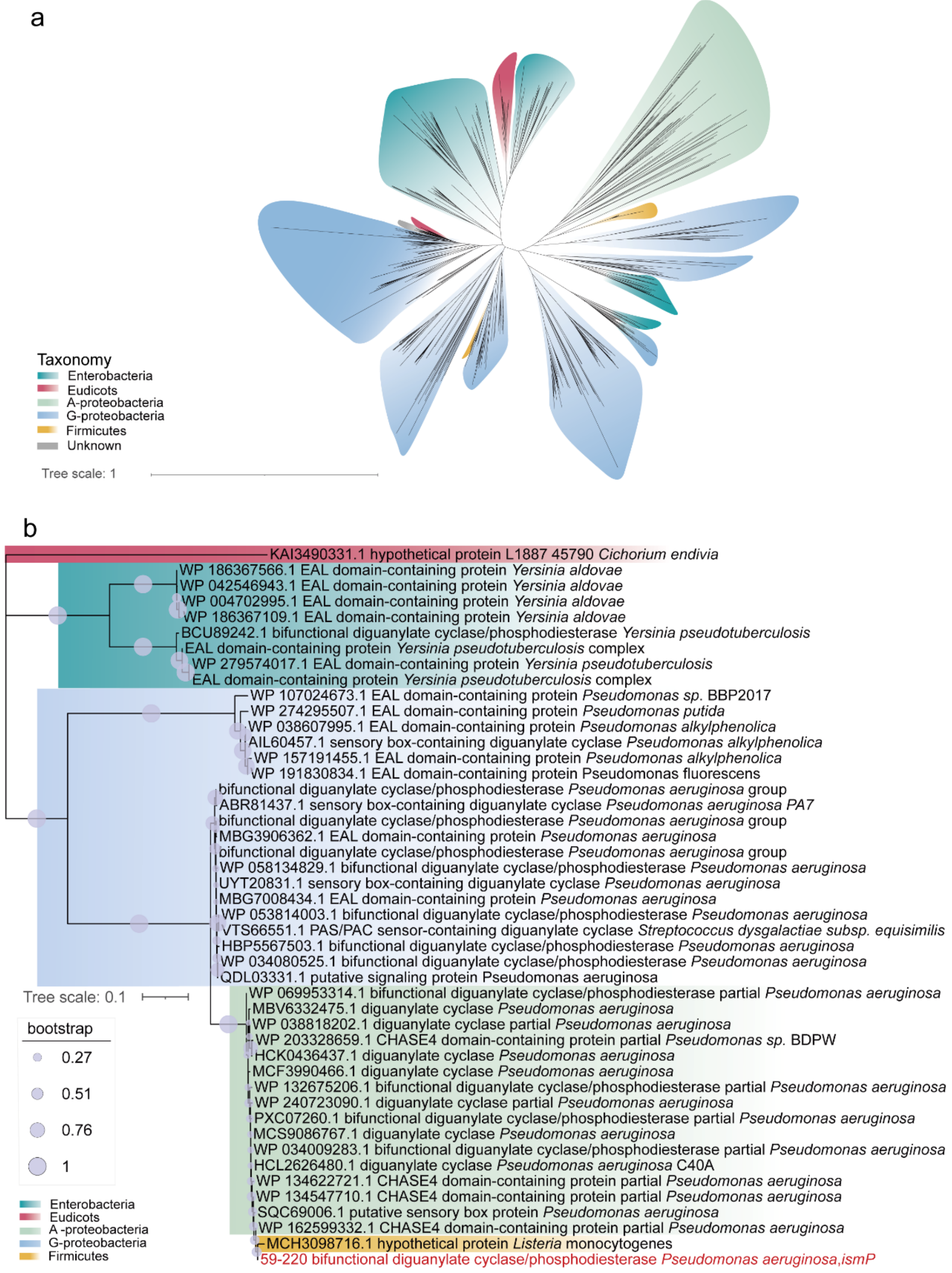

Supplementary Fig. 10 | Nearest-neighbour phylogenetic tree of CHASE4. a, Based on the sequence similarity of CHASE4, an unrooted Nearest-neighbour phylogenetic tree was built from an alignment of 1718 sequences with 470 amino acid positions. The findings revealed that this chemoreceptor was predominantly found in *Enterobacteriales*, *Pseudomonadales*, *Enterobacter*, *A-proteobacteria*, and *G-proteobacteria*. Despite being different bacterial genera, both were Gram-negative, aerobic rod-shaped bacteria that belonged to the *Proteobacteria phylum*. They had the potential to cause infections in humans and animals and possessed type III secretion systems and iron metabolism capabilities. Interestingly, the CHASE4 domain in these two bacteria was primarily located at the N-terminus of proteins containing EAL or GGDEF-EAL domains. This suggested a co-evolution of the CAHSE4 domain with its chemotaxis recognition. **b**, The rooted Nearest-neighbour phylogenetic was built by aligning 112 sequences with 470 amino acid positions. CHASE4 homologs with the highest protein homology (>50%), such as *Pseudomonas aeruginosa*, *Pseudomonas fluorescens*, *Pseudomonas alkylphenolica*, *Pseudomonas putida*, *Yersinia aldovae* etc., indicated that CHASE4 was widely distributed in some pathogenic microorganisms, especially opportunistic pathogens. Notably, only PA0847 and PA2072 in PAO1 contain the CAHSE4 domain, and their extracellular signals had been confirmed to bind iron ions in previous ITC experiments (Fig. 2c and Supplementary Fig. 4f). Given the wide distribution of CHASE4 homologs, it suggests that it may sense different extracellular chemical signals in different pathogenic microorganisms. Finally, in species with low homology (<45%) CAHSE4 homologs (such as *Cichorium endivia* and *Listeria monocytogenes*), we found that CHASE4 originated earliest in *Cichorium endivia*.

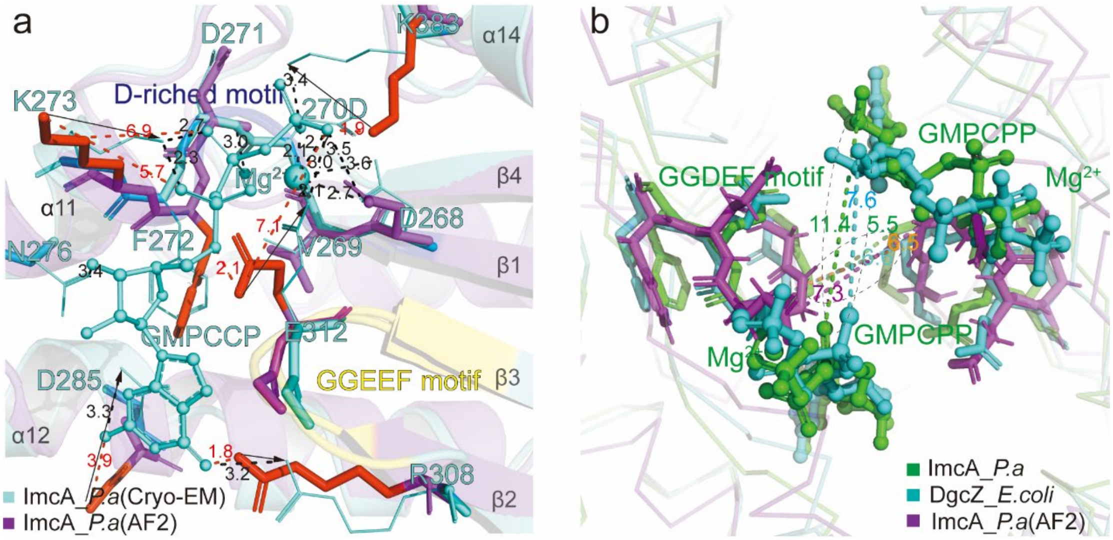

Supplementary Fig. 11 | a, The structural superposition of ligand-bound form with its corresponding apo-form of ImcA. Conformational changes of residue sidechains caused by the ligand binding to the ImcA homo-dimer are highlighted by red sticks with its shift direction indicated by black arrows, and corresponding distance to the ligand atom in red. **b**, The ribbon diagram of the comparison of ImcA dimeric DGC domain with relevant structures. The conserved GG(D/E)EF motifs are shown with sticks. And the substrate analog in ImcA from *P.a* and DgcZ from *E.coli*. are represented with green and cyan ball-and-stick, respectively.

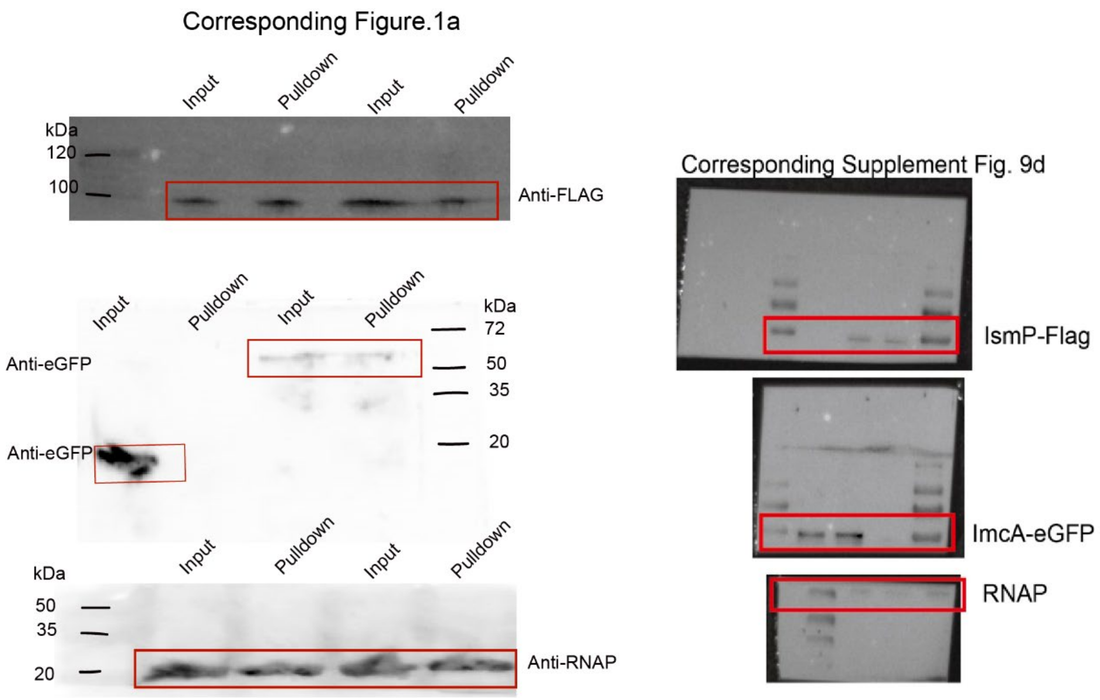

Supplementary Fig. 12 | Uncropped versions of all scans

151 **Supplementary Table 1 | Cryo-EM data collection, refinement, and validation statistics.**

	IsmP
**Data collection and processing**	
EM equipment	Titan Krios (Thermo Fisher Scientific)
Voltage (kV)	300
Detector	Gatan K3
Pixel size (Å)	0.668
Defocus range (μm)	-0.8 ~-3.0
Magnification	130,000 ×
Frames	32
[illegible]	55
Number of collected micrographs	14509
Number of selected micrographs	14408
Number of used particles	212874
Map Resolution (Å)	3.07
Symmetry	C2
**Refinement**	
Initial model	De-novo
Model Resolution (Å)	3.07
Model composition	
Chains	3
Non-hydrogen	5958
Residues	Protein: 757 Nucleotide: 0
Water	0
Ligands	Mg:2<br>G2P:4
B factors (Å^2)	
Protein	30.00/281.32/158.73*
Ligand	182.78/209.42/196.10*
Water	---
R.m.s. deviations	
Bonds (RMSD)	0.004
Bonds length (Å)	1.025
Validation	
MolProbity	1.72
Clashscore	12.17
Ramachandran plot statistics (%)	
Preferred	97.34
Allowed	2.66
Outlier	0.00

152 *Minimum/maximum/mean

153

Supplementary Table 2 | The X-ray crystallographic table of IsmP.

Data collection and Refinement	IsmP
Wavelength (Å)	0.9791
Resolution range (Å)	42.79-1.90 (1.95-1.90)
Space group	$P22_12_1$
Unit cell a b c (Å) α β γ (°)	64.43 75.20 104.08 90 90 90
Total reflections	336346 (14226)
Unique reflections	40448 (2842)
Multiplicity	11.4 (11.3)
Completeness (%)	99.67 (99.09)
Mean I/sigma(I)	17.65 (6.15)
Wilson B-factor (Å^2)	23.24
R_{merge}	0.104 (0.4339)
R_{meas}	0.1091 (0.4548)
R_{pim}	0.03257 (0.1351)
$CC_{1/2}$	0.998 (0.987)
CC^*	1 (0.997)
Reflections used in refinement	40448 (2842)
Reflections used for R-free	1973 (148)
R_{work}	0.2254 (0.2846)
R_{free}	0.2626 (0.3752)
CC(work)	0.197 (-0.034)
CC(free)	0.158 (0.069)
Number of non-hydrogen atoms	3670
macromolecules	3276
ligands	6
solvent	388
Protein residues	428
R.M.S.deviation (bonds) (Å)	0.007
R.M.S.deviation (angles) (°)	0.94
Ramachandran favored (%)	98.11
Ramachandran allowed (%)	1.89
Ramachandran outliers (%)	0
Rotamer outliers (%)	0
Clashscore	4.8
Average B-factor (Å^2)	29.72
macromolecules	29.04
ligands	20
solvent	35.66

156 **Supplementary Table 3 | C-di-GMP Metabolism Related Proteins Interacting with PA2072**
157 **Protein in model organism P.aeruginosa PAO1**

Protein	Enzymatic activity	Regulated functions or phenotypes	Feature
PA0861(RbdA)	DGC	Motility, production of rhamnolipids and EPS	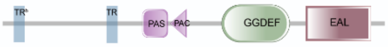
PA1120 (YfiN)	DGC	EPS production	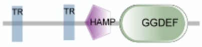
PA1851	ND[a]	ND	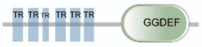
PA2072	ND	ND	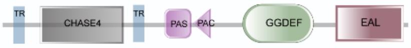
PA2200	PDE	T3SS gene expression,biofilm formation	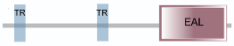
PA1433(LapD)	-	Surface adhesin production	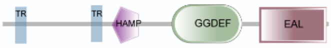
PA2870	DGC	ND	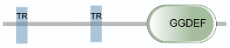
PA3825	PDE	T3SS gene expression,biofilm formation	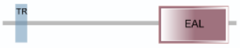
PA4367 (BifA)	PDE	Swarming motility and T4P function	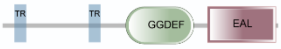
PA5017 (DipA or Pch)	PDE	EPS production,biofilm formation and dispersa	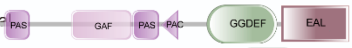
PA5442	ND	ND	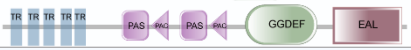

a.ND:not determined.
158 b.TR:transmembrane region.PAS/PAC/HAMP/CHASE4:signal sensor domain or signal transduction domain.

Supplementary Table 4. Strains and plasmids used in this study

Strain or plasmid	Relevant characteristics	Source
E. coli		
DH5α	*F – φ80lacZ ΔM15 Δ(lacZYA-argF) U169 recA1 endA1 hsdR17(rk, mk+) phoA supE44 thi-1 gyrA96 relA1 tonA*	Invitrogen
BL21 (DE3)	*F – ompT hsdSB (rB- mB-) gal dcm met* (DE3)	Invitrogen
C43 (DE3)	*F–ompT hsdSB (rB-mB-) gal dcm* (DE3)	Invitrogen
BTH101	*F-, cya-99, araD139, galE15, galK16, rpsL1 (Str ^{r}), hsdR2, mcrA1, mcrB1.*	Invitrogen
P. aeruginosa		
PAO1	Wild type	This lab
Δ*ismP*	*ismP* deletion mutant of PAO1	This study
Δ*imcA*	*imcA* deletion mutant of PAO1	This study
**plasmids**		
pAK1900	*E. coli–P. aeruginosa* shuttle cloning vector carrying Plac upstream of MCS; Cb^{r}	Lab stock
pAK1900-*ismP*	High-level expression vector utilizing the lac promoter, *ismP* cloned in pAK1900 vector	This study
pAK1900-*ismP*$^{E341A/A342R/R346A}$	High-level expression vector utilizing the lac promoter, pAK1900 containing *ismP* with Glu at 341 and Arg at 346 these amino acids mutated to Ala, Ala at 342 mutated to Arg	This study
pAK1900-*ismP*^{E341A}	High-level expression vector utilizing the lac promoter, pAK1900 containing *ismP* with Glu at 341 mutated to Ala	This study
pAK1900-*ismP*^{A342R}	High-level expression vector utilizing the lac promoter, pAK1900 containing *ismP* with Ala at 342 mutated to Arg	This study
pAK1900-*ismP*^{R346A}	High-level expression vector utilizing the lac promoter, pAK1900 containing *ismP* with Arg at 346 mutated to Ala	This study
pAK1900-*ismP*$^{L28R/G31R/W35R}$	High-level expression vector utilizing the lac promoter, pAK1900 containing *ismP* with Leu at 28, Gly at 31, Trp at 35, these amino acids mutated to Arg	This study
p*cdrA_lux*	*lux* based *cdrA* promoter reporter plasmid; Kan^{r} , Tmp^{r}	Lab stock
pET28a	T7 lac promoter-operator, N-terminal His tag; kan^{r}	Invitrogen
pEX18Amp	Gene replacement vector from pUC18; sacB^{+}Amp^{r}	Lab stock

pEX18Amp-*ismP*	*ismP* deletion plasmid, pEX18Ap with upstream and downstream region of *ismP*	This study
pEX18Amp-*imcA*	*imcA* deletion plasmid, pEX18Ap with upstream and downstream region of *imcA*	This study
pET28a-CHASE4_PASPAC	Protein expression construct, CHASE4_PASPAC cloned in pET28a vector	This study
pET28a-CHASE4	Protein expression construct, CHASE4 cloned in pET28a vector	This study
pET28a-*ismP*	Protein expression construct, *ismP* cloned in pET28a vector	This study
pET28a-*imcA*	Protein expression construct, *imcA* cloned in pET28a vector	This study
pET28a-CHASE4$^{E65A\ N66A\ Q69A}$	Protein expression construct, pET28a containing CHASE4 with Glu at 65, Asn at 66, Gln at 69, these amino acids mutated to Ala	This study
pET28a-CHASE4^{E65A}	Protein expression construct, pET28a containing CHASE4 with Glu at 65 mutated to Ala	This study
pET28a-CHASE4^{N66A}	Protein expression construct, pET28a containing CHASE4 with Asn at 66 mutated to Ala	This study
pET28a-CHASE4^{Q69A}	Protein expression construct, pET28a containing CHASE4 with Gln at 69 mutated to Ala	This study
pET28a-PA0847_CHASE4	Protein expression construct, PA0847_CHASE4 cloned in pET28a vector	This study
pRSFDuet2-*ismP*$_{His\_}$*imcA*	Protein expression construct, *ismp* and *imcA* cloned in pRSFDuet2 vector	This study
pME6032	High-level expression vector utilizing the tac promoter; Tc^{r}	This study
pME6032-*imcA*	High-level expression vector, *imcA* cloned in pME6032 vector	This study
pME6032-*imcA*$^{R223A\ F41A\ N45A}$	High-level expression vector, pME6032 containing *imcA* with Arg at 223, Phe at 41, Asn at 45, these amino acids mutated to Ala	This study
pME6032-*imcA*^{R26A}	High-level expression vector, pME6032 containing *imcA* with Arg at 26 mutated to Ala	This study
pKT25	Expressing the T25 fusion, Kan^{r}	Lab stock
pUT18C	Expressing the T18 fusion, Amp^{r}	Lab stock
pMini-CTX	Integration plasmid; Tc^{r}	Lab stock
pMini-CTX-*ismp*-FLAG	Integration plasmid; pMini-CTX containing *ismp*-FLAG	This study

pMini-CTX-*ismp*-FLAG-*imcA*-eGFP	Integration plasmid; pMini-CTX-*ismp*-FLAG containing *imcA*-eGFP	This study
pMMB67EH-eGFP	High-level expression vector utilizing the tac promoter, C-teminal eGFP tag; Cb^r	Lab stock
p-*imcA*-eGFP	High-level expression vector utilizing the tac promoter, pMMB67EH-eGFP containing *imcA*	This study
pMMB67EH-Flag	High-level expression vector utilizing the tac promoter, C-teminal Flag tag; Cb^r	Lab stock
pMMB67EH-*ismp*-Flag	High-level expression vector utilizing the tac promoter, pMMB67EH-Flag containing *ismP*	This study

Supplementary Table 5 | Primers used in this study

Name	Sequence	Application
KO2072F1	ATTGGATCCTCGGCGCTGGCGTTCAAGAT	constructing *ismP* deletion mutant
KO2072R1	AAATCTAGACTGCGGTACCGCTGGCAATC	
KO2072R2	AAAAAGCTTCCAGGGCACCAGGAAGTACA GCATC	
KO2072F2	ATTTCTAGAGTTTCCGCCGAAGGCCATGCT	
KO1851F1	ATTGGATCCGGTGCAGATCAGCGCGTC	constructing *imcA* deletion mutant
KO1851R1	ATTTCTAGAGCGAGTTCCAGCTGAGAAA	
KO1851R2	ATTAAGCTTAGGCGCTCGCGGGAGTACAC	
KO1851F2	ATTTCTAGACGCGCTCTACGAGGCCAAGG	
CHASE4f	ATTGGATCCGCCAGGCATCAGGACGACATC	Constructing protein plasmid
CHASE4r	AAGCTTTCACAGCAGGTGCAGGTTGGCG	
pET-CHASE4_PASPACf	GGATCCATCGCCAGGCATCAGGACG	
pET-CHASE4_PASPACr	AAGCTTCTCGTCGGTGATGTCGGCCG	
pRSF-IsmPf	GGATCCATGCTTGCTTCAGCGATTGCC	
pRSF-IsmPr	AAGCTTTCAAGGCCGGCGCGCAGCA	
pRSF-ImcAf1	AAGCTTTCAAGGCCGGCGCGCAGCA	
pRSF-*ImcA*r1	GAGGGTACCTTATTCCAGACGATCATGCTG GCGA	
pET-*ImcA*f	GGATCCAAGCTTTCACAGCAGGTGCAGGTT GGCG	
pET-*ImcA*r	AAGCTTCTATTCGAGGCGGTCGTGCTG	
pET-PA0847CHASE4F	ATTGGATCCCGTTTCGACCGAGAGGAC	
pET-PA0847CHASE4R	ATTAAGCTTGATGTAGAGCAGGCGTTTCTT G	
pME-*ImcA*$^{F41A\ N45A}$f	GCCCTCAGCTGGGCCTCGCTGATGCAGCGG CGCGCTGCCA	Constructing mutated overexpression plasmid
pME-*ImcA*$^{F41A\ N45A}$r	CGAGGCCCAGCTGAGGGCCTCGCGAAACT CGTTTTCCAGC	
pME-*ImcA*^{R223A}f	CTCGGCGCCCGGCTGCTGGGCATGATGGCC GAAC	
pME-*ImcA*^{R223A}r	GCAGCCGGGCGCCGAGGAACTGCTCGCGC TGGGCGTAGTC	
pME-*ImcA*^{R26A}f	AACGGCTTTGCTTGGTTGCGGTTCGAAAAG	
pME-*ImcA*^{R26A}r	CAACCAAGCAAAGCCGTTGCGCAACTGGC GCG	
pAK-*IsmP*$^{E341A\ A342R\ R346A}$f	CTAGATGGATCGGGGCTCGGCTGGACGAGT TGCTGGATAGCGATACGG	

pAK-*IsmP*$^{E341A\ A342R\ R346A}$r	CAGCCGagcCCCGATCCAtctagCCGGCTGGTAGCCGGTGACGTC	
pAK-*IsmP*^{A342R}f	CGGAGagaTGGATCGGGCGGCGGCTGGACGAG	
pAK-*IsmP*^{A342R}r	CGCCGCCCGATCCAtctCTCCGGCTGGTAGCCG	
pAK-*IsmP*^{E341A}f	CGGctGCCTGGATCGGGCGGCGGCTGGACGAG	
pAK-*IsmP*^{E341A}r	CCCGATCCAGGCagCCGGCTGGTAGCCGGTGACGTC	
pAK-*IsmP*$^{L28R/G31R/W35R}$f	CagaGCCCTCagaGCATGCGGCagaGCGATCCTGCACATCGCCAGGCATC	
pAK-*IsmP*$^{L28R/G31R/W35R}$r	GCATGCTCTGAGGGCTCTGAAAAGGGCGGCCAGGAACATTAGGAGATGCTTGG	
p-*imcA*-eGFP f	attGGATCCATGCTTGCACGCGACAGC	
p-*imcA*-eGFP r	attGGTACCTCATTCGAGGCGGTCGTG	
pET-CHASE4$^{E65A/N66A/Q69A}$f	GCTGCCTCCGAGGCCttCTCCACCACCTATTCCTTCTGGACC	Constructing mutated protein plasmid
pET-CHASE4$^{E65A/N66A/Q69A}$r	AGGCGGCCTCGGAGGCAGCCCGGCGATTCTGCAGCGC	
pET-CHASE4^{E65A}f	GCCGGGCTAATTCCGAGCAGTTCTCCACCACC	
pET-CHASE4^{E65A}r	GCTCGGAATTAGCCCGGCGATTCTGCAGCG	
pET-CHASE4^{N66A}f	GAAGCCTCCGAGCAGTTCTCCACCACC	
pET-CHASE4^{N66A}r	ACTGCTCGGAGGCTTCCCGGCGATTCTGCAGCG	
pET-CHASE4^{Q69A}f	AGCAGGCCTCCACCACCTATTCCTTCTGGACCG	
pET-CHASE4^{Q69A}r	GTGGTGGAGGCCTGCTCGGAATTTTCCCGGCG	
Mini-CTX-*imcA*-eGFP f1	ATTGGTACCTGGCACAACCTCATGGCGAT	Integration plasmid
Mini-CTX-*imcA*-eGFP r1	ctcgcccttgctcaccatTTCGAGGCGGTCGTGCTG	
Mini-CTX-*imcA*-eGFP f2	atggtgagcaagggcgag	
Mini-CTX-*imcA*-eGFP r2	CGGCGGCTCTCAGggtaccTCActtgtacagctcgtcca	
Mini-CTX-*ismp*-FLAG f	attGGTACCCTGAGAGCCGCCGCGCGC	
Mini-CTX-*ismp*-FLAG r	attGGATCCAGGCCGGCGCGCAGCAT	

Supplementary References

1. Strop P, Brunger AT. Refractive index-based determination of detergent concentration and its application to the study of membrane proteins. *Protein Sci* **14**, 2207-2211 (2005).
