# Supplementary material for: A c-di-GMP signaling module controls responses to iron in Pseudomonas aeruginosa
Source: Nat Commun. 2024 Feb 29;15:1860. doi: 10.1038/s41467-024-46149-3 (PMC10904736; doi:10.1038/s41467-024-46149-3)
Supplement: Supplementary file 3 — Reporting Summary [file 41467_2024_46149_MOESM3_ESM.pdf]

nature portfolio

Corresponding author(s): Haihua Liang

Last updated by author(s): Feb 14, 2024

Reporting Summary

Nature Portfolio wishes to improve the reproducibility of the work that we publish. This form provides structure for consistency and transparency in reporting. For further information on Nature Portfolio policies, see our Editorial Policies and the Editorial Policy Checklist.

Statistics

For all statistical analyses, confirm that the following items are present in the figure legend, table legend, main text, or Methods section.

n/a	Confirmed	
☐	☒	The exact sample size (*n*) for each experimental group/condition, given as a discrete number and unit of measurement
☐	☒	A statement on whether measurements were taken from distinct samples or whether the same sample was measured repeatedly
☐	☒	The statistical test(s) used AND whether they are one- or two-sided<br>*Only common tests should be described solely by name; describe more complex techniques in the Methods section.*
☒	☐	A description of all covariates tested
☒	☐	A description of any assumptions or corrections, such as tests of normality and adjustment for multiple comparisons
☐	☒	A full description of the statistical parameters including central tendency (e.g. means) or other basic estimates (e.g. regression coefficient) AND variation (e.g. standard deviation) or associated estimates of uncertainty (e.g. confidence intervals)
☐	☒	For null hypothesis testing, the test statistic (e.g. *F*, *t*, *r*) with confidence intervals, effect sizes, degrees of freedom and *P* value noted<br>*Give P values as exact values whenever suitable.*
☒	☐	For Bayesian analysis, information on the choice of priors and Markov chain Monte Carlo settings
☒	☐	For hierarchical and complex designs, identification of the appropriate level for tests and full reporting of outcomes
☒	☐	Estimates of effect sizes (e.g. Cohen's *d*, Pearson's *r*), indicating how they were calculated

Our web collection on statistics for biologists contains articles on many of the points above.

Software and code

Policy information about availability of computer code

Data collection	The data of the evolutionary tree comes from NCBI. The prediction results of protein secondary structure are derived from SMART.
Data analysis	Evolutionary tree analyses are performed using MEGA11. The biofilm content is quantified with Image J. For processing LC-MS data, we employ Xcalibur (version 4.0), Image J (version 1.5.4), and Trace Finder (version 4.1). The MST data procured were subsequently analyzed using MO. Offinity Analysis (X86). The data from ITC were analyzed using NanoAnalyze Software (v.3.11.0).

For manuscripts utilizing custom algorithms or software that are central to the research but not yet described in published literature, software must be made available to editors and reviewers. We strongly encourage code deposition in a community repository (e.g. GitHub). See the Nature Portfolio guidelines for submitting code & software for further information.

Data

Policy information about availability of data

All manuscripts must include a data availability statement. This statement should provide the following information, where applicable:

- Accession codes, unique identifiers, or web links for publicly available datasets
- A description of any restrictions on data availability
- For clinical datasets or third party data, please ensure that the statement adheres to our policy

Source data are provided with this paper. The cryo-EM density maps for ImcA have been deposited in Electron Microscopy Data Bank (EMDB) under the accession code EMD-37444 (https://www.ebi.ac.uk/emdb/search/EMD-37444). And the corresponding atomic coordinate has been deposited in the Protein Data Bank (PDB) under the accession code 8WCN (https://www.rcsb.org/structure/8WCN). Meanwhile, the atomic coordinate of IsmP homodimer crystal structure has been

deposited PDB under the accession code 8WCT (https://www.rcsb.org/structure/8WCT). Prediction models used as starting point for modelling IsmP-ImcA oligomer were deposited in AlphaFold Protein Structure Database under accession codes: Q9I243 (https://alphafold.com/entry/Q9I243) for IsmP, Q9I2P4 (https://alphafold.com/entry/Q9I2P4) for ImcA.

Research involving human participants, their data, or biological material

Policy information about studies with human participants or human data. See also policy information about sex, gender (identity/presentation), and sexual orientation and race, ethnicity and racism.

Reporting on sex and gender	N/A
Reporting on race, ethnicity, or other socially relevant groupings	N/A
Population characteristics	N/A
Recruitment	N/A
Ethics oversight	N/A

Note that full information on the approval of the study protocol must also be provided in the manuscript.

Field-specific reporting

Please select the one below that is the best fit for your research. If you are not sure, read the appropriate sections before making your selection.

[x] Life sciences [ ] Behavioural & social sciences [ ] Ecological, evolutionary & environmental sciences

For a reference copy of the document with all sections, see nature.com/documents/nr-reporting-summary-flat.pdf

Life sciences study design

All studies must disclose on these points even when the disclosure is negative.

Sample size	Experiments were performed with a minimum of three independent replicates (n=3 or 4, as indicated in the figure legends). The sample size was determined based on standard experimental designs in this field, without a pre-calculation of the sample size. A typical sample size of 3 or 4 was chosen to ensure sufficient statistical power and to minimize random variations. This sample size is consistent with common practice in the field (Cui and Zhang et al., 2022).
Data exclusions	No data was excluded.
Replication	Reproducibility was ensured by at least 3 independent replicates.
Randomization	In our experiment, we employed a randomization method for sample selection. Specifically, we randomly selected three or four bacterial colonies from a solid plate for subsequent testing. This random selection process helps ensure the reliability of our experimental results by minimizing potential bias caused by sample selection. We did not specifically control for any covariates, as we believe that all colonies grow under the same conditions in our experimental design, thus eliminating the need for additional covariate control. Our randomization method is based on standard practices in our field to ensure fairness and reproducibility of the experiment.
Blinding	In our experiment, blinding was not applicable. This is because our data collection and analysis were automated, without any manual counting or scoring. We randomly selected three or four bacterial colonies from a solid plate for subsequent testing, a process that is random and not influenced by any expected outcomes. Therefore, our experimental results are fair and objective, without the need for blinding.

Reporting for specific materials, systems and methods

We require information from authors about some types of materials, experimental systems and methods used in many studies. Here, indicate whether each material, system or method listed is relevant to your study. If you are not sure if a list item applies to your research, read the appropriate section before selecting a response.

Materials & experimental systems

n/a	Involved in the study
☐	☒ Antibodies
☒	☐ Eukaryotic cell lines
☒	☐ Palaeontology and archaeology
☒	☐ Animals and other organisms
☒	☐ Clinical data
☒	☐ Dual use research of concern
☒	☐ Plants

Methods

n/a	Involved in the study
☒	☐ ChIP-seq
☒	☐ Flow cytometry
☒	☐ MRI-based neuroimaging

Antibodies

Antibodies used

Mouse anti DDDDK-Tag mAb (AE005, ABclone, Wuhan, China); Rabbit anti GFP-Tag pAb (AE011, ABclone, Wuhan, China);The RNAP antibody was gifted by a colleague. Rabbit anti-Mouse IgM mAb (AS094, ABclone, Wuhan, China); Goat Anti-Rabbit IgG (AS014, ABclone, Wuhan, China)

Validation

https://abclonal.com.cn/catalog/AE005
https://abclonal.com.cn/catalog/AE011
https://abclonal.com.cn/catalog/AS094
https://abclonal.com.cn/catalog/AS014

Plants

Seed stocks

N/A

Novel plant genotypes

N/A

Authentication

Describe any authentication procedures for each seed stock used or novel genotype generated. Describe any experiments used to assess the effect of a mutation and, where applicable, how potential secondary effects (e.g. second site T-DNA insertions, mosiacism, off-target gene editing) were examined.
